# Complete genome sequence of *Serratia plymuthica* EIF4, a prodigiosin-producing isolate from freshwater

**DOI:** 10.1128/mra.00557-26

**Published:** 2026-07-10

**Authors:** Ines Friedrich, Hannah Bornemann, Jana Wagner, Vasundhara Karthikeyan, Anja Poehlein, Rolf Daniel

**Affiliations:** 1 Department of Genomic and Applied Microbiology, University of Göttingen 9375, Göttingen, Germany; University of Manitoba, Winnipeg, Canada

**Keywords:** *Serratia plymuthica*, prodigiosin, pigmented bacteria

## Abstract

We present the complete genome sequence of the prodigiosin-producing bacterium *Serratia plymuthica* EIF4 isolated from freshwater in Osterode am Harz, Germany. The genome comprises a 5,539,881-bp chromosome and a 91,684-bp plasmid with guanine-cytosine contents of 55.84 and 52.36%, respectively.

## ANNOUNCEMENT

**Fig 1:**
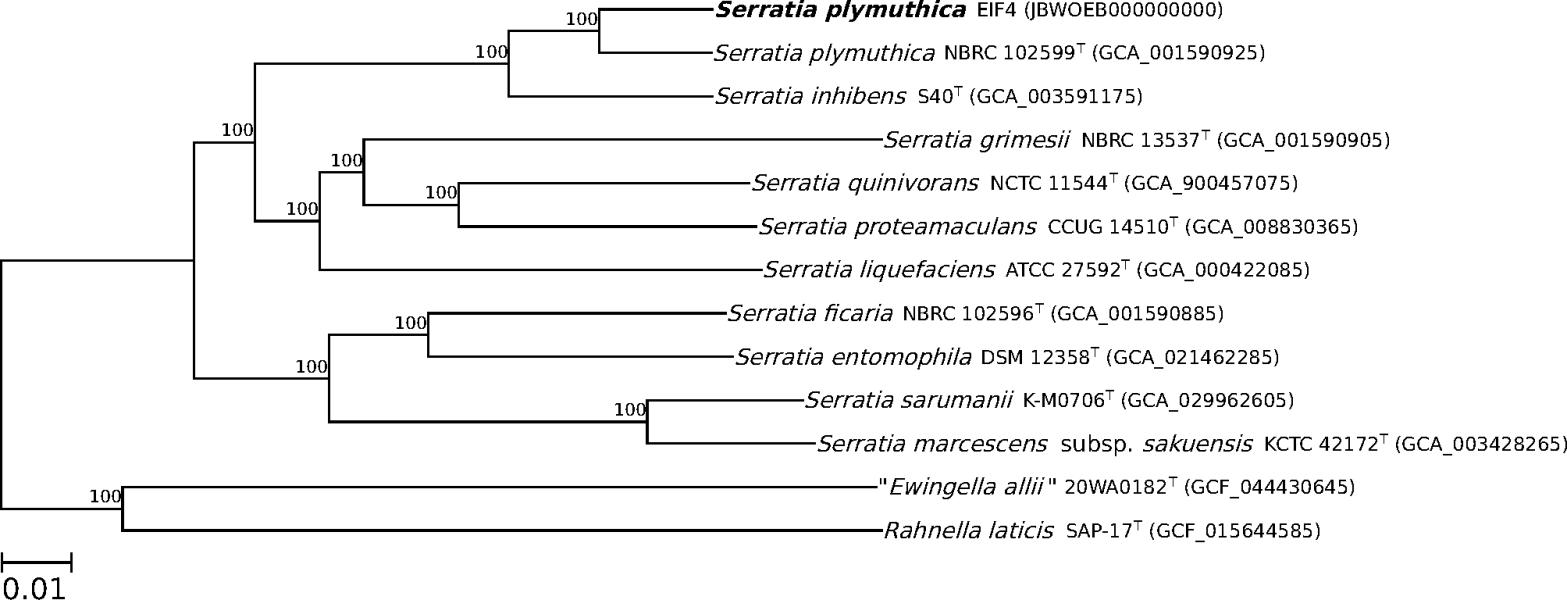
Whole-genome phylogenetic tree showing the position of *Serratia plymuthica* EIF4 (bold) among closely related *Serratia* type strains. The tree was inferred by using TYGS (14). The tree was calculated from GBDP distances using FastME with 100 pseudo-bootstrap replicates (15). The tree was visualized using ETE 3 (16).

Members of the genus *Serratia* are known producers of the pigment prodigiosin (1). Here, we report the complete genome sequence of strain EIF4 isolated from freshwater of the Bremke River near Osterode am Harz, Germany (51°44′36.7″ N, 10°16′06.4″ E) on 12 December 2023 to expand the genomic representation of environmental *Serratia* strains. To enrich pigmented bacteria, 1 mL of river water was applied to cooked long rice in a Petri dish (100 mm × 15 mm) and incubated at 25°C following the method described by Corpe (2). After 2 days, rice grains exhibiting red pigmentation were transferred to R2A agar plates (3). A red-pigmented isolate was purified by repeated streaking on R2A agar and designated EIF4. A colony was grown overnight in R2A broth at 25°C and 180 rpm. Cells were harvested by centrifugation, and high-molecular weight DNA was extracted using the Monarch HMW DNA Extraction Kit for Tissue according to the manufacturer’s instructions (New England Biolabs, Ipswich, MA, USA). Whole-genome sequencing was performed using Oxford Nanopore sequencing (Oxford Nanopore Technologies, Oxford, UK). For library preparation, 600 ng of genomic DNA was processed using the ligation-based Native Barcoding Kit 24 V14 (SQK-NBD114.24; barcode 22) following the manufacturer’s instructions. Genomic DNA was neither sheared nor size-selected prior to library preparation. Sequencing was performed for 92 h on a MinION Mk1B equipped with an R10.4.1 flow cell and controlled by MinKNOW v24.02.06. Default parameters were used for all software, unless otherwise noted. Base calling and demultiplexing were performed using Dorado v0.8.0 (SUP model). Adapter and barcode sequences were trimmed during demultiplexing. Sequencing generated 556,392 reads totaling 1,677,456,993 bp, with a read N50 of 6,812 bp. Reads shorter than 1 kb or of low quality were removed using Filtlong v0.3.1 (4). Filtered reads were subsampled with Trycycler v0.5.4 (5) based on an estimated genome size of 5.5 Mb. Independent assemblies were generated with Flye v2.9.5 (6), Raven v1.8.3 (7), and Miniasm v0.3 (8) employing Minipolish v0.1.3 (7). Assemblies were combined using Trycycler, and the consensus assembly was polished with Medaka v2.0.0 (Oxford Nanopore Technologies). The chromosome and plasmid sequences were reoriented using Dnaapler v1.2.0 (9) to start at the *dnaA* gene and a predicted replication initiation gene (*repA*), respectively. Reads were mapped to the final assembly using Minimap2 v2.29 (10), and coverage was calculated with SAMtools v1.22 (11), yielding an average coverage of 257-fold. The final assembly consisted of two circular contigs: a chromosome of 5,539,881 bp with a guanine-cytosine (GC) content of 55.84% and a plasmid of 91,684 bp with a GC content of 52.36%. Genome completeness was estimated using CheckM2 v1.1.0 (12). Genome annotation was performed using the National Center for Biotechnology Information Prokaryotic Genome Annotation Pipeline (PGAP) v6.10 (13). Assembly statistics and genome features are summarized in Table 1. Phylogenomic analysis using the Type (Strain) Genome Server (TYGS) (14; accessed 04 April 2026) assigned EIF4 to *S. plymuthica*. The dDDH value (formula d4) to *S. plymuthica* NBRC 102599^T^ was 72.7%, exceeding the 70% species threshold (Fig. 1). EIF4 represents a novel strain of *S. plymuthica*.

**TABLE 1:** General features of the genome of *Serratia plymuthica* EIF4 (accession JBWOEB000000000) based on PGAP annotation (13) and CheckM2 analysis (12).

Feature	Data
Total number of genes	5,332
Number of protein-coding sequences	5,211
Number of rRNA genes	
5S	8
16S	7
23S	7
Number of tRNA genes	86
Number of ncRNA genes	13
Number of pseudogenes	83
CRISPR arrays	1
Genome completeness	100%
Genome contamination	0.2%

## Data Availability

The complete genome sequence has been deposited in DDBJ/ENA/GenBank under accession number JBWOEB000000000. The associated BioProject and BioSample accession numbers are PRJNA1233243 and SAMN47263980, respectively. Raw nanopore reads are available in the NCBI Sequence Read Archive under accession number SRR37797452.
